# Advancing Robotic Single-Site Cholecystectomy: Innovations, Challenges, and Future Directions

**DOI:** 10.7759/cureus.60643

**Published:** 2024-05-20

**Authors:** Ubaid Ansari, Burhaan Syed, Romteen Sedighi, Khadija Ansari, Muzammil Akhtar, Yinka Davies

**Affiliations:** 1 Neurology, California Northstate University College of Medicine, Elk Grove, USA; 2 Radiology, California Northstate University College of Medicine, Elk Grove, USA; 3 Internal Medicine, California Northstate University College of Medicine, Elk Grove, USA; 4 Internal Medicine, Mission College, Santa Clara, USA; 5 Surgery, California Northstate University College of Medicine, Elk Grove, USA; 6 Gastroenterology, California Northstate University College of Medicine, Elk Grove, USA

**Keywords:** future direction, implications, limitations, cholelithiasis, robotic single-site cholecystectomy, cholecystectomy, laparoscopic surgery

## Abstract

The crystalization of the components of bile within the gallbladder can lead to the formation of gallstones (cholelithiasis), which may often require surgical removal of the gallbladder, a procedure known as cholecystectomy, in symptomatic cases. Robotic single-site cholecystectomy (RSSC) is a recently introduced groundbreaking minimally invasive procedure for gallbladder removal. RSSC utilizes robotic technology, offering enhanced dexterity through a single-incision approach, promising improved outcomes such as reduced postoperative pain and superior cosmesis. However, certain limitations, such as restricted instrument movement and heightened hernia risk, necessitate a critical evaluation of this modality. Furthermore, as the widespread adoption of RSSC remains undecided due to concerns over its costs, efficiency, and overall superiority over prior models, this paper assesses future possibilities for RSSC's evolution. In vivo robotics, improved digital imaging, and re-engineering of the surgical instruments themselves are all potential avenues to augment the current RSSC design, although it is currently unclear as to what extent they could impact the procedure’s viability. This review critically examines the available literature on the effectiveness and potency of RSSC compared to its predecessors in the modern healthcare setting and proposes future directions through which innovation could more firmly establish the procedure as the standard of care for cholecystectomy.

## Introduction and background

Gallstones, or cholelithiasis, refer to hardened deposits of bile that accumulate in the gallbladder. There are many types of gallstones including cholesterol stones, pigment type stones, and mixed stones. Previous research highlights that 90% of cholelithiasis patients have cholesterol stones [1]. Ever since the first abdominal incision for cholecystitis with calculi in 1684 till the invention of the cholecystectomy procedure in 1882, many advancements were made in the surgical management of cholelithiasis [2]. In 1987, laparoscopy was first used in medicine, and later in 1991, robots were incorporated [3]. The single-incision laparoscopy (first performed in 1997), the multiple-port robotic-assisted laparoscopic cholecystectomy, and most recently, the single-port robotic-assisted laparoscopic cholecystectomy pioneered in 2010 are the newest advancements in biliary surgery [4]. In this review, we explore robotic single-site cholecystectomy (RSSC), a relatively novel procedure that is quickly gaining appeal in both research and clinical settings, by evaluating recent studies in the literature.

RSSC is an emerging technique in minimally invasive surgical procedures, revolutionizing the landscape of gallbladder removal. The advent of robotic technology has transformed traditional laparoscopic modalities by offering enhanced dexterity, precision, and a single-incision approach, promising improved patient outcomes and reduced surgical trauma [5]. Using RSSC results in patients having reduced postoperative pain, less need for additional postoperative analgesics, and better cosmetic results compared to the traditional multiport method. However, RSSC also restricts the orientation and range of motion of the instrument arms in the single incision site, rendering some movements more challenging and increasing the risk of postoperative hernia due to bigger single incisions [6]. Amidst its promising advancements, a critical evaluation of RSSC is imperative to identify its limitations, understand its economic implications, and pave the way for its future trajectory in the field of surgery.

The economic implications of implementing RSSC merit considerable attention. While the initial investment in robotic technology is itself substantial, understanding the long-term economic impact, including cost-effectiveness, resource utilization, and reimbursement issues, becomes pivotal in assessing the feasibility and sustainability of integrating RSSC into routine surgical practice [7]. Furthermore, exploring the future direction of RSSC is fundamental to assessing its potential enhancements and advancements. This review will delve into emerging technologies, procedural refinements, and ongoing research avenues aimed at overcoming the current limitations and optimizing the utility of RSSC. Predicting and envisioning the course of the advances in RSSC in the context of evolving surgical innovations and technological breakthroughs will be pivotal in shaping its future landscape.

In summary, this review aims to dissect the multifaceted aspects of RSSC, shedding light on its limitations, dissecting its economic implications, and delineating the potential future directions. By elucidating these critical facets, this review endeavors to contribute to informed decision-making among healthcare professionals and stakeholders, ultimately steering the trajectory of RSSC toward greater efficacy, accessibility, and patient-centered care.

## Review

Robotic single-site cholecystectomy

While a cholecystectomy has traditionally been performed using laparoscopy, thanks to recent medical breakthroughs, the procedure can now be augmented via the da Vinci® Surgical System robot technology. The first robotic-assisted laparoscopic procedures were performed in the early 1990s and the first single-site robotic cholecystectomy study was carried out on pigs. These procedures did not involve external robotic arm collisions or technological issues [8]. According to a pilot study, the same procedure with robotic aid required less time to set up on average compared to its non-robotic counterpart and had no additional adverse side effects [9]. One study employed the GelPort® technology, which docks numerous da Vinci® laparoscopic ports into a single tiny incision, to perform the RSSC on pigs [10], and found that the technique could be performed successfully, safely, and with little blood loss without switching to an open approach. On average, the surgery lasted 70 minutes. By utilizing a gel seal cap, the GelPort® design with a single incision offers dependable pneumoperitoneum management [10].

The first RSSC on a human was conducted in April 2010 by Kroh et al., and the next 13 cases studied afterward were all successful with no intraoperative problems (Table 1). The benefits of robotic assistance include fewer incisions and scars, a quicker recovery time, improved visualization of the surgical field, greater precision, and a lower chance of harming surrounding structures [4]. Several recent papers and medical professionals have raised concerns about the overall complications of robotic surgery. Although laparoscopy can be performed with one port, the single-site port (22.5-centimeter vertical incision at the umbilicus) is better suited for a robotic approach thanks to the single port’s capacity for laparoscopic instruments, the availability of a camera, and room for instrument movement without interference [4].

**Table 1:** Summary of studies on robotic single-site cholecystectomy RSSC: Robotic single-site cholecystectomy

Author	Country	Objective	Type of study	Main points
Ponsky (2011) [9]	United States	To determine the feasibility of RSSC	Comparative study	The same surgical procedure with robotic assistance was faster than its non-robotic alternative, on average; there were no outstanding morbidities in the robotic version
Sugimoto et al. (2011) [10]	Japan	To use the GelPort laparoscopic system to evaluate the functionality and technical aspects of RSSC for hepatobiliary surgery in a preliminary animal study	Comparative study	The procedure was safe and had minimal blood loss; there was no need to transition to an open operation; 70 minutes was the standard run time of the whole procedure; the GelPort laparoscopic system is beneficial in its maintenance of the pneumoperitoneum by using a gel seal cap
Kroh et al. (2011) [4]	United States	To assess the results of an RSSC using the da Vinci single-site platform on a human	Experimental study	The first RSSC performed was done without any intraoperative complications; the da Vinci® robotic system uses more ergonomic instruments that have improved range of motion; the da Vinci® system provides a clearer view via 3-dimensional viewing and magnification; this robotic approach is more expensive, time-consuming, and has a learning curve
Konstantinidis et al. (2012) [11]	Greece	To utilize a new platform from Intuitive Surgical to analyze the feasibility, safety, and efficacy of RSSC	Experimental study	RSSC implements flexible endoscopic robotic arms that prevent instruments from clashing during the procedure; the procedure lasted 84.5 minutes on average; few postoperative complications occurred, but one postoperative hemorrhage was noted; patients went home within 24 hours, happy with their results; the equipment was useful for patients with high BMIs
Ricciardiello et al. (2020) [12]	Italy	To determine if RSSC can achieve minimization of surgical trauma compared to the gold standard laparoscopic cholecystectomy	Comparative study	The mean operation time was 99.6 ±21.5 minutes; no abdominal drain was needed for any patients; the presence of incisional hernias at the site of insertion for the single-port noted for 3 patients; patients who underwent trans-umbilical incisions were more likely to experience incisional hernias; patients believed a robotic surgery to be superior to an alternate method due to its cosmetic benefits
Jang et al. (2021) [13]	South Korea	To present a single surgeon’s initial experiences in RSSC	Experimental study	RSSC procedures in this study had a mean operative time of 39.3 ±12.5 minutes; the docking time decreased significantly just after two cases; one patient suffered from a wound seroma a day following the operation; the surgeon experienced no learning curve despite never using RSSC previously

Alternatively, the da Vinci® robotic system uses more ergonomic tools with greater ranges of motion, thereby allowing triangulation to be restored by separating the tools beyond the port entry and at the working end. As a result, once intraperitoneal, the procedure closely resembles the usual laparoscopic cholecystectomy more than the single-port laparoscopic method. Additionally, the da Vinci® system's three-dimensional vision, magnification, and tremor-suppressing capabilities enable clearer visibility, making cholecystectomy safer for the patient on average [4]. The downside is that this robotic approach is more expensive, more time-consuming, and has a learning curve. This has raised doubts about RSSC offering any benefits that make it superior to the current treatment.

An early study involved 45 patients and investigated their experience with RSSC using da Vinci® with the Single-Site® platform (Table 1). By utilizing endoscopic, dynamic robotic arms, the da Vinci® Single-Site® platform prevented instruments from clashing in individual cases. Although there was a single postoperative hemorrhage, there were no further accounts of technical issues, open or multiport conversions, or major intraoperative complications [11]. On average, the operation time amounted to 84.5 minutes. Within 24 hours, typically, patients were discharged and sent home. These patients were reportedly happy with the procedure and its results in the following two months. Overall, perioperative pain was minimal and subjects were able to resume normal daily activity in just around four and a half days [11]. Additionally, the employed equipment was highly useful in patients with higher BMIs.

Another study of 27 consecutive patients who underwent RSSC procedures for cholecystolithiasis at the SS. Annunziata Hospital in Chieti, Italy (Table 1) aimed to analyze the intraoperative and postoperative data of patients to critically assess the technical practicality and cosmetic outcomes. One patient with acute cholecystitis who underwent an open procedure for four days was excluded from the final analysis. The mean operative time was 99.6 ±21.5 minutes and no intra- or postoperative complications and readmissions were reported [12]. No abdominal drain was required in any of the procedures, indicating the absence of infection, inflammation, or injury, which typically accumulate fluid in the abdomen. However, doctors recorded the presence of an incisional hernia at the site of insertion for the single-site port in three patients. These incisional hernias were discovered in patients who experienced trans-umbilical incisions, but not in those with peri-umbilical incisions [12].

In the future, peri-umbilical incision with detachment of the umbilical scar should be considered for RSSC instead of trans-umbilical incision to prevent incisional hernias, even though further research is needed on this approach. According to the Body Image Questionnaire results for one-year follow-ups, patients had positive perceptions of their bodies and their scars. Moreover, patients' self-rating of the scar considerably improved when they were informed of the outcomes and shown postoperative images of alternative surgical techniques. At the end of the survey, 25 out of 26 patients reported that robotic surgery was preferable to alternative surgical procedures due to superior cosmetic outcomes [12]. These findings indicate that RSSC may be favored for patients who struggle with body image perception and have more advanced cosmetic needs.

Another study discussed the experience of 74 patients who underwent RSSC between April 2019 and August 2020 at the Dong-A University Medical Center in Busan, South Korea (Table 1). One surgeon, who had extensive expertise performing laparoscopic surgery but had never before done RSSC, performed these procedures [13]. In a systematic review comparing 13 papers with data on 1010 patients who underwent RSSC for benign gallbladder disease, the mean total operative time was 77.29 minutes. However, RSSC procedures in this study had a mean total operative time of 39.3 ±12.5 minutes and a mean docking time of 7.6 ±3.1 minutes, suggesting that a highly experienced surgeon may not be impeded when performing robotic surgery for the first time [13]. In addition, the operation and docking time decreased as the surgeon gained more experience, cutting the time in half after two cases. The robotic approach may be quite intuitive and may not have a steep learning curve, contrary to prior concerns regarding RSSC. A single patient in this study had a serious postoperative complication following RSSC, requiring two weeks of antibiotics and dressing for wound seroma [13].

Limitations and economic implications of RSSC

A comparative study of RSSC and conventional laparoscopic cholecystectomy was conducted to determine the difference in cost and operation time between the two procedures. Fifty consecutive patients who underwent RSSC were compared to fifty patients who underwent CLC, taking into account age, gender, ASA score, histology, and surgical experience [14]. It was found that operative time and hospital stay time were similar between the two procedures; however, overall hospital costs were notably higher for RSSC at $7985.4 (SD 1760.9) compared to CLC at $6255.3 (SD 1956.4) (p<0.001). This difference was mainly attributed to the amortization and consumables associated with the robotic system [14]. Considering the higher cost of RSSC and the minimal benefits over CLC, it may not be pertinent for hospitals and clinics to switch completely to RSSC; however, a case-by-case financial study would likely need to be done to assess this.

In another study, a cost-benefit analysis was conducted on the da Vinci robot to determine if it was worth utilizing the machine with its high costs. The machine was purchased for $1.5 million with a $112,000 service contract per year and $200 per case for disposables [15]. Laparoscopic radical prostatectomy (LRP) and robotic-assisted radical prostatectomy (RAP) were used in the analysis. With the machine being amortized, along with service contract fees, the cost of the robot amounted to $415,00 a year. To maintain profits, simply switching from LRP to RAP cases is not enough; there must be an increase in the number of RAP cases. Institutions with a high amount of LRP cases can convert to RAP and maintain profits, however, to make up for the lower volume of LRP cases, a greater number of RAP cases must be performed to maintain profits [15]. This indicates that the purchase of the da Vinci robot may not be financially viable in institutions with a low volume of LRP procedures. Cost-benefit analysis still needs to be done with cholecystectomies, and case-by-case financial studies must be performed to properly assess the benefit the machine will bring in for a specific institution. 

Although it possesses many benefits, RSSC comes with certain limitations. As elaborated upon earlier, the single-site laparoscopic cholecystectomy is a scarless surgery with minimal access. Nevertheless, further studies with both larger patient sample sizes and longer terms of research are quite necessary to endorse the use of RSSC, and to fully validate it above its predecessors. Even still, the smaller studies conducted thus far may provide enough evidence to support the lack of harm from the RSSC with the use of more resources. When considered in its entirety, robotically assisted laparoscopy is regarded as both safe and satisfactory for patients, particularly given its aesthetic outcomes without scars. The RSSC is a safe procedure and, in Sugimoto’s conclusive words, “enormously advantageous to the patient”. Furthermore, four out of five surgeons who performed several RSSCs were eager to explore robotic technology for various other surgical types [16]. Robotic technology is reckoned as a technique that will add significant value to performing complex hepatobiliary surgeries [8].

Yet, despite the many advantages, it has been found that multiple surgical instruments deployed through a single port make triangulation manipulation more difficult and increase the possibility of instrument clashes. Such traits could very well lead to surgeon fatigue and frustration [4]. In fact, one study concluded that a more flexible robot arm would be required to perform single-incision surgery more comfortably [9]. Other factors such as decreased freedom of movement, fewer ports that can be used, longer docking time leading to longer operation time, and the proximity of the instruments to each other during the operation are all valid concerns regarding RSSC [17]. However, before changes can be implemented, the matter of limitations should be thoroughly investigated regarding the procedures in different fields. The robotic single-port procedures have been conducted in urology and gynecology, and hence the matter of what individual issues the field is facing must be taken into account before changes are made to the arm as a whole. Potential ideas and proposals to overcome the limitations should be investigated as and when offered [4].

Future directions

The latest advancement in this sphere may involve the utilization of miniature in vivo robots [18]. These multidextrous but small robots enable better manipulation and efficiency of usage, with the added benefits of lower rates of morbidity and reduced cost concerns [18]. It has been experimented on three pigs. In vivo robot users have reported better robot arm flexibility, triangulation without collision, and increased visualization [18]. The in vivo robots may be manipulated either by telepresence or remote control. Active efforts are underway to decrease the size of the robots with aspirations to potentially deploy more than one robot arm per case to perform various tasks [18].

Other advancements include improvements in the computer technology and image processing technology of the da Vinci® surgical system robot technology [19]. In one study, 65 patients underwent radical distal gastrectomy with the da Vinci® surgical system, and 89 patients underwent radical distal gastrectomy with traditional laparoscopy. A thread image edge detection algorithm was implemented for the robotic surgeries, which yielded significantly higher sensitivity, specificity, and accuracy for images that were relayed to the surgeon [19]. Improvements in optics and imaging for the da Vinci® surgical system may transform the equation between the surgeon and the machine, giving the surgeon a better understanding of what is happening in the patient’s body at the time of the procedure. The performance of the machine’s optics and vision are continuously improving, and work is being done to further improve the computing aspect of the machine, such as start and running speed, as well as image processing techniques [19].

Considering the current limitations of the RSSC model, improvements can be made to further refine this procedure. One factor that can be improved pertains to the set of instruments used in laparoscopic surgeries. The common instruments used are reusable 5-mm laparoscopic graspers, laparoscopic scissors, a single-use dissection hook, and the occasional trocar to drain unexpected fluid [20]. Despite the benefits of single-site laparoscopic surgery, one main concern regarding the procedure is the difficulty of triangulation of the instruments, as well as instrument clashing due to single-site incisions. To bypass this limitation, one innovation could be to create instruments with more articulations or movable joints. Making instruments that can mimic the movements of the human arm can make the procedure more effective, leading to fewer problems with triangulation and instrument placement. Studies must be conducted with more flexible and bendable instruments to determine the effect they could have on triangulation errors and surgeon frustration. 

Another innovation that could tackle the problem of instrument clashing is to shrink and combine instruments. Miniaturizing the surgical instruments can allow for better ergonomics and maneuverability, as it makes more space available within the abdomen to work without clashing. Another option for reducing instrument clashes would be to reduce the number of instrument insertions in the incision. This could be done by combining instruments so that there is less crowding around the site of the incision. One such combination could be a laparoscopic grasper and trocar. Instead of having a separate insertion for the trocar, having them combined can lessen the crowding of the instruments as well as retain both functions. Again, further research must be done to determine the impact smaller instruments and combined tools will have on ergonomics and instrument clashing. 

Limitations and economic implications of a new model

However, such improvements to the existing models of laparoscopic surgery are not without their limitations. Augmenting the number of articulations for each instrument undoubtedly increases the complexity of operational use, which may result in an extended learning curve for physicians before surgical competence is achieved. The docking and overall procedure times of the instruments may also be adversely affected as the complexity of the machinery, tools, and digital enhancements increases. Cost is an additional consideration; as discussed previously, the more modern RSSC is already associated with a significantly higher expenditure than traditional laparoscopy, both due to the initial investment into da Vinci units and the refilling price of consumable parts. It stands to reason that an even more advanced apparatus utilizing in vivo robotics, computer-enhanced optics, and higher precision multi-purpose arms would face similar if not higher barriers, including manufacturing costs, that have weakened the appeal of RSSC in terms of cost-benefit analyses.

Additionally, decreasing instrument size to heighten surgical precision and avoid clashing may lead to greater device fragility, which could put patients at risk of postoperative infection if fragments are lost in the body, simultaneously incurring a human cost and contributing to further repair concerns. The combination of the mechanical arms to facilitate the range of motion may also prove inadequate if critical instruments are then unable to be deployed concurrently when needed, resulting in interference with different surgical tasks. Ultimately, while innovation in the ergonomics and sustainability of RSSC remains the decisive path forward in the evolution of laparoscopy, further research on the engineering and programming of the surgical infrastructure, in addition to improved training methods and affordability, will be necessary to ensure that new generation devices are viable.

Summary

In recent years, the field of minimally invasive surgery has witnessed remarkable advancements, with RSSC emerging as a practical alternative to traditional laparoscopy and single-incision cholecystectomy for the treatment of gallbladder disease. This review has explored the evolution of cholecystectomy techniques, focusing on RSSC, its benefits, drawbacks, and future directions. Historically, cholecystectomy has evolved from open surgical procedures to laparoscopy, and finally to robotic-assisted techniques. RSSC, facilitated by the da Vinci® Surgical System, has garnered attention for its potential to offer patients reduced postoperative pain, improved cosmetic outcomes, and shorter hospital stays. However, it is essential to weigh these advantages against the limitations of RSSC. One of the primary challenges of RSSC is the constraint on instrument movement within the single incision site, leading to difficulties in triangulation and instrument clashes. Additionally, the higher cost, longer procedure times, and steeper learning curve associated with robotic technology have raised concerns related to its cost-effectiveness. Despite these limitations, the evidence suggests that RSSC can be a safe and reasonable procedure for cholecystectomy, especially for patients with uncomplicated gallbladder disease who prioritize cosmetic results and shorter recovery times. 

Looking to the future, innovations in laparoscopic instruments, such as more flexible and miniature tools, hold promise for overcoming the limitations of RSSC. These advances may enhance ergonomics, reduce instrument clashes, and improve overall surgical precision. Furthermore, the integration of in vivo robots and advancements in computer technology and imaging processing within the da Vinci® surgical system may further refine the RSSC procedure. However, it is crucial to acknowledge that with these innovations come new challenges, including increased complexity, extended learning curves, and the potential fragility of smaller instruments. Additionally, the economic implications of adopting these advanced technologies must be carefully considered in the context of healthcare budgets and cost-effectiveness.

## Conclusions

RSSC represents a significant advancement in the field of cholecystectomy, offering distinct benefits for patients with uncomplicated gallbladder disease. While it is not without limitations and there exist concerns related to cost-effectiveness, ongoing research and technological innovations hold the potential to further enhance the feasibility and effectiveness of RSSC. As the field continues to evolve, surgeons, researchers, and healthcare institutions must collaborate to navigate these challenges and ensure that patients receive the best possible care while minimizing the impact of gallbladder disease on their lives.
